# Multimodal brain-computer interface communication in disorders of consciousness

**DOI:** 10.1186/1471-2202-15-S1-P118

**Published:** 2014-07-21

**Authors:** S Halder, I Käthner, A Kübler

**Affiliations:** 1 Institute of Psychology, University of Würzburg, 97070 Würzburg, Germany

## 

Brain-computer interfaces (BCIs) can provide a means of communication for people with severe motor impairments [1]. For people with traumatic or other brain injuries establishing communication has proven to be more problematic [2]. The main challenge is integrating a mechanism to deal with co-occurring disorders of consciousness.

This abstract describes multimodal (auditory, tactile, visual) P300 BCI communication attempts performed with a 42-year-old male who had an intracranial hemorrhage of the cerebellum and brainstem one year prior to the measurements. The participant is paralyzed except for residual eye blinking and movement as well as some movement of the left hand. This residual muscular control is used for communication (termed conventional).

Many pitfalls were encountered when attempting to establish communication. The main aspect is that when we performed the same task at different time points the results varied considerably (see Figure 1).

**Figure 1:**
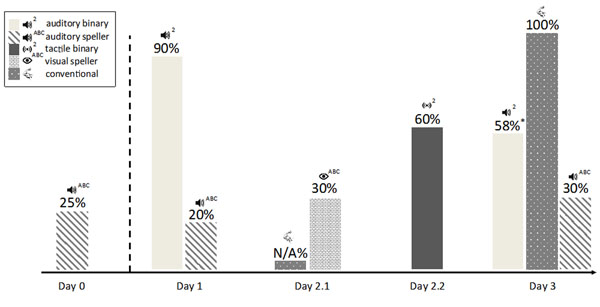
Attempts performed to establish communication with the locked-in participant. Day zero was performed as an initial testing two months before the other measurements. The height of the bars indicates the offline classification rates (BCI with linear discriminant analysis, conventional with expert rated videos).

## Conclusions

Albeit the fact that the attempts with the brain-computer interface was not successful in establishing free communication, the classified responses were clearly above chance. This illustrates that the user understood the instructions and has the physiological capacities to generate the responses required to operate the system. The fluctuations of vigilance experienced by patients with disorders of consciousness must be taken into account when developing new generations of BCI systems.
